# Incidence of Gonadal and Extragonadal Germ Cell Tumours in Patients With Klinefelter Syndrome

**DOI:** 10.1111/andr.70208

**Published:** 2026-03-06

**Authors:** Aksh Tailor, Oishee Bandyopadhyay, Nahian Ahmed, Kapishan Shanmugathasan, Alice Cotton, ChingHao Chen, Tet Yap

**Affiliations:** ^1^ GKT School of Medical Education King's College London London UK; ^2^ Department of Urology Guy's and St Thomas’ NHS Foundation Trust London UK

**Keywords:** c‐KIT, cryptorchidism, extragonadal germ cell tumour, hypergonadotropic hypogonadism, Klinefelter syndrome, mediastinal tumour, primordial germ cells, testicular descent, testicular germ cell tumour

## Abstract

**Background:**

Klinefelter's syndrome (KS; 47, XXY) is associated with an altered risk profile for malignancies compared with non‐KS males. In particular, several reports have noted a striking association between KS and extragonadal germ cell tumours (EGCTs), especially in the mediastinum, whereas the risk of testicular germ cell tumours (TGCTs) in KS remains unclear. KS patients also have a higher prevalence of cryptorchidism (undescended testes)—a known risk factor for TGCT in the general population—yet it is uncertain if cryptorchidism confers the same cancer risk in the KS population. This study aims to compare the incidence of TGCTs and EGCTs in KS against the general population, and to evaluate the impact of cryptorchidism on TGCT risk in KS.

**Methods:**

We conducted a retrospective cohort study of 233 KS patients (age range 2–83, mean 35.1 years) managed at a dedicated KS clinic between 2019 and 2025. Clinical histories, ultrasound findings and histopathology reports were reviewed to identify cases of TGCT, EGCT and cryptorchidism. Incidence rates per 100,000 patient‐years were calculated based on 8311 total person‐years of follow‐up (from birth until age of diagnosis or until end of study period—30th June 2025) and compared with population data from cancer registries. A one‐sided Poisson test was used to assess differences in observed tumour incidence versus the general population, with 95% confidence intervals (CIs) derived by exact Poisson methods.

**Results:**

In 233 KS patients, we identified two cases of TGCTs (both testicular tumours in adult men) and one case of mediastinal EGCT. The incidence of TGCT in the KS cohort was 24.1 per 100,000 patient‐years (95% CI, 3.0–88.6), which was higher than that in the general male population (∼4.8 per 100,000) but did not reach statistical significance (*p* = 0.060). In contrast, the incidence of mediastinal EGCT in KS was 12.0 per 100,000 patient‐years, exceeding the general population rate (∼0.04 per 100,000). The age‐standardised incidence ratios for TGCT was 2.24 (95% CI, 0.27–8.10) compared with the UK population. For EGCT, the crude age‐standardised incidence ratio was 83.0 (95% CI, 2.1–460.5). Cryptorchidism was documented in 10 of the 233 KS patients (4.3%). Notably, none of the KS patients with a history of cryptorchidism developed TGCT, and neither of the two KS‐TGCT patients had cryptorchidism or other typical risk factors for testicular cancer other than markedly atrophic testes.

**Discussion:**

KS confers a markedly increased risk of mediastinal EGCT but not a clear increase in TGCT. The pathogenesis of germ cell tumours in KS is multifactorial: aberrant primordial germ cell migration/survival and an altered hormonal milieu are central hypotheses for the predilection towards midline extragonadal tumours, whereas intrinsic testicular degeneration in KS might protect against TGCT despite risk factors like cryptorchidism and atrophy. Genetic factors (e.g., extra X chromosome effects, KIT mutations) and environmental influences (e.g., hypergonadotropic state) may also play roles in these observations.

**Conclusion:**

For clinicians managing Klinefelter patients, these findings emphasise the importance of vigilance for EGCTs, particularly in the mediastinum, even though routine testicular cancer surveillance beyond standard care may not be necessary in the absence of other risk factors. Our data do not support a need for intensive TGCT screening in KS at present, given the lack of a statistically significant increase in testicular cancer incidence. Conversely, the significantly elevated incidence of mediastinal GCT in KS raises the question of screening such as periodic chest imaging in adolescent KS patients. While universal screening is debatable due to the rarity of these tumours, a thorough physical exam (looking for mediastinal mass effects) and a low threshold for chest imaging if symptoms arise (e.g., chest pain, dyspnoea) are advisable in KS. In future, larger multi‐centre cohort studies and cancer registry linkages (ideally capturing genotype‐confirmed KS cases) will be needed to definitively quantify TGCT risk in KS and to explore the molecular underpinnings of the KS–GCT connection.

## 1 Introduction

Klinefelter's syndrome (KS) is the most common sex chromosome aneuploidy in males, occurring in approximately one in 600 live‐born males and typically characterised by a 47, XXY karyotype. The condition results in dysgenesis of the testes leading to hypergonadotropic hypogonadism (elevated luteinising hormone [LH] and follicle‐stimulating hormone [FSH] with low testosterone levels) and a constellation of clinical features: tall stature, small firm testes with infertility, eunuchoid body proportions, gynaecomastia and often learning or behavioural difficulties [1]. KS has been associated with an altered spectrum of malignancies compared with karyotypically normal men. Notably, epidemiological studies have found increased incidences of breast cancer, non‐Hodgkin lymphoma and mediastinal germ cell tumours (GCTs) in KS patients, whereas the overall cancer incidence in KS is similar to the general male population [2]. This paradox underscores the need to delineate which specific cancers are truly overrepresented in KS and which are not.

GCTs are the most common malignant tumours of the testes, accounting for ∼95% of testicular neoplasms in young adult men [3]. Testicular GCTs (TGCTs) comprise two main histologic categories: seminomas and non‐seminomas (the latter including embryonal carcinoma, yolk sac tumour, choriocarcinoma and teratoma, or mixtures thereof. In rare cases, GCTs arise outside the gonads, typically along the midline of the body (e.g., the mediastinum, retroperitoneum, pineal gland or sacrococcygeal region). These are termed extragonadal germ cell tumours (EGCTs). EGCTs are uncommon in the general population—representing roughly 2%–5% of all GCTs in adults—and their incidence is on the order of 0.1 per 100,000 males per year [4]. Intriguingly, primary mediastinal GCTs (particularly mediastinal non‐seminomatous GCTs) have long been observed to occur with disproportionately high frequency in KS patients [2]. It is estimated that 50%–70% of mediastinal GCTs in adolescent and young adult males occur in the setting of undiagnosed KS with some experts recommend karyotype screening for any male patient who presents with a mediastinal GCT.

By contrast, it has been debated whether TGCT risk is meaningfully elevated in KS. On one hand, KS patients often have risk factors that might predispose to testicular cancer: up to one‐quarter or more of KS males have a history of cryptorchidism (undescended testes) [5, 6], which in the general population confers a 3–10 times increased risk of TGCT depending on whether and when orchidopexy is performed [7]. Infertility is another known risk factor for TGCT in 46, XY men, and virtually all KS men are infertile. On the other hand, the testes in KS are typically small and poorly developed, often lacking the normal complement of germ cells by adulthood. Prior registry studies have not conclusively shown an excess of testicular cancer in KS—for example, a Danish cohort (696 KS men) in the 1990s observed no increase in TGCT incidence relative to expected rates [8]. Thus, prevailing literature suggests that KS is strongly associated with extragonadal (mediastinal) GCTs, but not clearly associated with higher TGCT incidence.

Given the limited data and somewhat contradictory notions, this study set out to clarify the incidence of both gonadal and extragonadal GCTs in KS using a contemporary single‐centre KS cohort to calculate incidence rates of TGCT and EGCT and compared these to population‐based cancer registries. We also examined the incidence of cryptorchidism in our KS cohort and the occurrence of TGCT among those with cryptorchidism, to test the hypothesis that cryptorchid KS patients might be an ‘at‐risk’ subgroup. Ultimately, our goal is to present a comprehensive, up‐to‐date analysis of GCT incidence in KS and to offer insight into the biological underpinnings.

## 2 Materials and Methods

### 2.1 Study Design and Population

We conducted a retrospective cohort study of KS patients seen at a Klinefelter Syndrome Multidisciplinary Clinic (London, UK) from 2019 through 2025. A total of 233 patients with genetically confirmed KS were included (seven paediatric cases under 18, and 226 adults). The mean age was 35.1 years (standard deviation 12.6; range 2–83 years). Paediatric cases were retained in this study as the comparative registries had also incorporated paediatric population data in their calculations. This clinic cohort is a tertiary referral population, and thus may somewhat enrich for patients with KS‐related complications. Ethical approval for this study was granted by the Guy's and St Thomas’ NHS Foundation Trust Institutional Review Board (IRB reference 18206). No written consent has been obtained as there is no patient identifiable data included in this study.

### 2.2 Data Collection

Each patient's medical records were reviewed for demographic information and clinical history, specifically focusing on known risk factors for testicular cancer. We recorded any history of cryptorchidism (undescended testis, either unilateral or bilateral, and whether it was surgically corrected), infertility, prior malignancies and any testicular lesions noted on examination or imaging. All patients underwent a detailed physical examination by an endocrinologist or urologist as part of their clinic visits, and the presence of cryptorchidism or testicular atrophy was documented.

Specialist radiologists with expertise in KS performed or supervised scrotal ultrasound examinations for adult patients. Ultrasound reports were reviewed to identify indeterminate intratesticular lesions (e.g., microcalcifications, small hypoechoic nodules) that might warrant further evaluation for GCT, as well as to measure testicular volumes (as an indicator of testicular atrophy). Standard B‐mode ultrasound and colour Doppler was used. Patients were scanned using the Philips EPIQ Elite system with a broadband linear array transducer of frequency 18–5 MHz. Where available, serum tumour markers (alpha‐fetoprotein, beta‐HCG, LDH) and pathology reports were obtained for any patient with a testicular mass or other clinical suspicion of GCT. Tumour marker data were available for 207 patients. Of the 26 with no tumour marker data, there was low clinical suspicion of malignancy.

### 2.3 Outcomes

The primary outcomes were the incidence of TGCT and EGCT in the KS cohort. TGCT was defined as any histologically confirmed GCT arising in the testis (including seminoma, non‐seminomatous GCT or mixed GCT). EGCT was defined as a GCT arising outside of the gonads, with no concurrent testicular tumour identified (*note*: if a metastatic GCT was found with an occult testicular primary, it would be considered a misclassified TGCT, but no such cases occurred in our cohort). For EGCT, we particularly noted the site (mediastinal, retroperitoneal, etc.)—in practice, all EGCT cases in this study were mediastinal. Secondary outcomes included the prevalence of cryptorchidism in the KS cohort, as well as any occurrence of TGCT among the cryptorchid subset.

### 2.4 Incidence Calculations

We calculated person‐years of follow‐up for each patient from birth until either the diagnosis of a GCT or the end of the study period (June 2025), whichever came first. For those without GCT, age at last follow‐up (or age in 2025) was used. The sum of person‐years for all 233 patients constituted the denominator for incidence rate calculations. Incidence rates for TGCT and EGCT were expressed per 100,000 patient‐years. Standardised incidence ratios (SIRs) were calculated as the ratio of observed to expected cases. For TGCTs, expected cases were obtained by multiplying cohort person‐years by male age‐specific incidence rates from Cancer Research UK [9]. For EGCTs, UK age‐specific rates were unavailable, therefore crude SIRs were derived using the overall male EGCT incidence rates reported by Arora et al. (2012). Exact Poisson 95% confidence intervals (CIs) were computed for these incidence estimates. Out of the 233 patients, there was no data regarding US findings for 21 cases. These patients were still involved in incidence calculations and contributed to the overall patient follow‐up years. To contextualise these rates, we gathered comparative incidence data for the general population and for KS from prior studies. Specifically, we used: (a) population‐based cancer registry data from England (1979–2003) reported by Arora et al. (2012) [4] for general male GCT incidence, and (b) data from a Danish cytogenetic registry study of KS patients (first January 1943 to 31st December 1991) by Hasle et al. (1995) [8] for KS‐specific incidence. Both registries involved adult and paediatric populations. As the general population‐based cancer registry included both female and male data, only male data were included in our calculations. Grouping of the GCTs was based on the following ICD‐9 and ICD‐10 codes by Arora et al. (2012): testicular (ICD‐9 site codes 186.0–187.9; ICD‐10 site codes C62.0–C63.9), mediastinum (ICD‐9 site codes 191.0–192.9, 194.3, 194.4, 225.0–225.9, 227.3, 227.4, 237.0, 237.1, 237.5 and 237.6; ICD‐10 site codes C71.0–C72.9, C75.1–75.3, D33.0–D33.9, D35.2–D35.4, D43.0–43.9, D44.3–D44.5). Grouping of GCTs was based on the following ICD‐7 codes by Hasle et al. (1995): testicular (178) and mediastinal (164). We also incorporated data reported by Rusner et al. (2013) [10] based on nine cancer registries from Germany between 1998 and 2008 and a nationwide Danish cohort by Bojesen et al. (2006) [11]. Differences between observed KS cohort cases and expected cases based on general population rates were assessed with one‐sided Poisson tests (reflecting the a priori hypothesis that incidence might be higher in KS). All statistical analyses were performed using R (R Foundation for Statistical Computing, Vienna, Austria). The design and reporting of this study adhered to the STROBE (Strengthening the Reporting of Observational Studies in Epidemiology) guidelines.

## 3 Results

### 3.1 Incidence of Germ Cell Tumours in KS Versus Non‐KS Populations

A total of 8311 patient‐years of follow‐up were calculated with a median of 35.0 and IQR of 18.4. The range of follow‐up years was 81.70 years (2.40–84.10). We identified two cases of TGCT and one case of EGCT.

Table 1 summarises the incidence rates observed in our KS cohort alongside comparative figures from prior studies.

**TABLE 1:** Incidence of germ cell tumours in KS and non‐KS populations (per 100,000 person‐years).

Tumour type	Incidence—Non‐KS (general population)	Incidence—KS (Hasle et al. 1995)	Incidence—KS (our cohort)
**Testicular GCT**	4.84 per 100,000 [4]	4.19 per 100,000	24.1 per 100,000 (95% CI, 3.0–88.6)
**Extragonadal GCT (all sites)**	0.145 per 100,000 [4]	21.0 per 100,000	12.0 per 100,000 (mediastinal only)
– **Mediastinal EGCT**	0.0430 per 100,000 [4]	16.8 per 100,000	12.0 per 100,000 (one case)

KS data are from our KS Clinic cohort (*n* = 233). Non‐KS data are from a population‐based English registry study (1979–2003). For reference, incidence in a historic Danish KS is also given.

TGCT incidence was 24.1 per 100,000 person‐years, which numerically exceeds the general population incidence of ∼4.8 per 100,000 [4]. However, this difference did not reach statistical significance owing to the small number of TGCT cases (two observed vs. ∼0.4 expected). The age‐standardised incidence ratios for TGCT was 2.24 (95% CI, 0.27–8.10) based on two observed cases and 0.89 expected cases according to Cancer Research UK age‐specific data. The 95% CI for our KS TGCT incidence was broad (3.0–88.6 per 100,000), reflecting the uncertainty with only two events. Both TGCT cases in KS were in adult patients (ages 37 and 36 years) and both presented as unilateral testicular masses that were identified through routine screening. One was a Stage IIA non‐seminomatous GCT (mixed embryonal and yolk sac histology) in the right testis, and the other was a seminoma (Stage I) in the right testis. Both patients underwent radical orchiectomy; the non‐seminoma case received adjuvant chemotherapy (bleomycin, etoposide, cisplatin) given lymphovascular invasion, whereas the seminoma case received a single cycle of carboplatin. Both are currently in remission having completed 4 years of recurrence‐free surveillance. Neither patient had a history of undescended testes, testicular microlithiasis or familial testis cancer. Notably, both had markedly small testes (< 4 mL volume) consistent with KS, and both had been on exogenous testosterone therapy prior to their cancer diagnoses. LDH was raised in the case of the seminoma (578), however, no tumour markers were raised in the non‐seminomatous GCT. In contrast, the EGCT incidence in KS was significantly elevated. We observed one primary mediastinal GCT (in a 20‐year‐old KS patient who presented with chest pain and was found to have an anterior mediastinal mass on imaging). Pathology revealed a mixed GCT (teratoma with embryonal carcinoma components). No tumour markers were raised in this patient. He was treated with surgical resection and chemotherapy and has no evidence of recurrence at 2‐year follow‐up. This single case translates to an incidence of 12.03 per 100,000 person‐years for mediastinal EGCT in our KS cohort, which is over two orders of magnitude higher than the incidence of mediastinal GCT in the general male population (∼0.04 per 100,000) [4]. The overall crude SIRs using population rates from Arora et al. (2021) for EGCT were 83.0 (95% CI, 2.1–460.5) and 279.8 (95% CI, 7.2–1560) for mediastinal EGCTs. Our finding is consistent with the Danish KS study by Hasle et al. (1995), which reported a mediastinal GCT incidence of 16.8 per 100,000 in KS [8], and with more recent analyses such as Williams et al. (2018) who noted a ∼67‐fold relative risk for mediastinal GCT in young KS males [12]. Out of the 233 patients, 113 (48.9%) adults underwent semen analysis. Of those, 108 (95.6%) had confirmed azoospermia. Semen analysis data were not available for the seven paediatric cases.

### 3.2 Cryptorchidism and Testicular Cancer Risk in KS

Cryptorchidism (undescended testis) was documented in 10 out of 233 KS patients in our cohort, yielding a cryptorchidism prevalence of 4.29%. Of these, seven had unilateral cryptorchidism and three had bilateral. All but one were corrected in childhood via orchidopexy (one patient with unilateral cryptorchidism was diagnosed in adulthood upon referral and declined surgery due to testicular atrophy). The cryptorchidism rate in our KS population is lower than some earlier reports which ranged from 27% up to 37% [5]. Those higher figures often come from small series or studies focusing on paediatric KS patients; our cohort, which includes many adults diagnosed later in life, might under‐estimate congenital cryptorchidism prevalence due to recall bias or incomplete documentation from infancy. A Danish registry study by Bojesen et al. found that KS males had a 6.25‐fold higher hazard of cryptorchidism compared with chromosomally normal males [11] corroborating that cryptorchidism is indeed significantly more frequent in KS. In the general male newborn population, cryptorchidism occurs in about 1%–3% at 1 year of age (after spontaneous postnatal descent in many cases) [7, 13]. The ∼4% observed in our adult KS cohort likely reflects those who had persistent cryptorchidism requiring surgical correction (or remained undescended).

Crucially, none of the 10 KS patients with cryptorchidism in our cohort developed a TGCT. This zero‐incidence persisted despite long follow‐up into adulthood for most of them (cumulative ∼300 patient‐years among the cryptorchid KS subset). Similarly, Accardo et al. (2014) reported no testicular tumours in a series of 40 KS patients, 11 of whom had a history of cryptorchidism [14]. These findings stand in stark contrast to the well‐established relationship between cryptorchidism and testicular cancer in otherwise healthy males. For context, Table 2 compares TGCT incidence in cryptorchid patients with and without KS.

**TABLE 2:** Incidence of testicular germ cell tumour among patients with cryptorchidism: KS versus non‐KS.

Patient group	Incidence of TGCT (per 100,000 person‐years)	Source/study
KS patients with cryptorchidism	0 (no cases in *n* = 10; 95% CI, 0–118)	Current study
KS patients with cryptorchidism	0 (no cases in *n* = 11)	Accardo et al. (2014) [14]
Non‐KS with cryptorchidism	45.5 per 100,000	Swerdlow et al. (1997; UK cohort, *n* = 1075) [15]
Non‐KS with cryptorchidism	26.7 per 100,000	Pettersson et al. (2007; Sweden cohort, *n* = 16,983) [7]

Historical cohort studies have quantified the testicular cancer risk in men with a history of undescended testes. A British cohort of 1075 cryptorchid boys (followed from 1951 into adulthood) found a TGCT incidence of 45.47 per 100,000 person‐years equating to roughly a 4.5% lifetime risk [15]. A larger Swedish registry study observed a somewhat lower incidence (∼26.7 per 100,000) among men who had orchidopexy, with risk strongly dependent on the age at surgery (those corrected after puberty had much higher cancer rates than those fixed in early childhood) [7]. In general, cryptorchidism is associated with a three to eightfold increased relative risk of TGCT in non‐KS individuals [16, 17]. Therefore, given that cryptorchidism is more frequent in KS, one might expect KS men to have higher TGCT rates. However, our data and the literature do not bear this out: KS effectively ‘uncouples’ cryptorchidism from testicular cancer. The absence of TGCT in cryptorchid KS patients is a striking observation that suggests additional protective or mitigating factors are at play, as discussed later.

## 4 Discussion

This study evaluated the incidence and distribution of gonadal and EGCTs in patients with KS, with additional analysis of how cryptorchidism interacts with TGCT risk in this unique population. The findings support a growing consensus that KS confers a markedly elevated risk of mediastinal (extragonadal) GCTs, while the association with TGCTs remains far less clear and may not be significantly increased. Furthermore, cryptorchidism in the KS population does not appear to amplify TGCT risk to the same extent observed in 46, XY males.

One of the central findings was the substantially elevated incidence of mediastinal GCTs in the KS cohort—12.0 per 100,000 person‐years compared with approximately 0.043 per 100,000 in the general population of males [4, 8]. This result aligns with historical studies, such as Hasle et al. (1995), who documented a 67‐fold increased incidence of mediastinal GCTs in KS men [8]. Similarly, Williams et al. (2018) found that approximately one‐third of males with primary mediastinal non‐seminomatous GCTs had KS, estimating a 19‐fold overall increase in GCT risk among KS individuals, with a particularly strong predisposition to mediastinal involvement [8, 12]. Strikingly, in children under age 15 with mediastinal GCTs, KS was present in over 80% of male cases [17, 18]. We did not find any cases of intracranial GCTs (e.g., pineal germinomas) in our KS patients. The mediastinum appears to be the dominant extragonadal site for GCTs in KS, consistent with aggregated data from case series and reviews (in KS, ∼70%–80% of extragonadal GCTs occur in the mediastinum, with the remainder mostly in other midline sites like sacrococcygeal or pineal regions) [17, 18]

In contrast, the relationship between KS and TGCTs appears less definitive. Although this study observed a TGCT incidence of 24.1 per 100,000—about five times the general male rate of 4.8–6 per 100,000—the association did not reach statistical significance [4]. This suggests that any increase in TGCT risk among KS men is modest at best. Previous population‐based studies support this finding. The Danish KS cohort reported no testicular cancers, despite approximately 0.3 being expected [8], and Bojesen et al. (2006) similarly found no excess TGCT risk in KS men [11]. These data collectively support a site‐specific susceptibility: KS greatly increases the risk of mediastinal GCTs but does not appear to significantly predispose to gonadal tumours.

All TGCT cases in KS that we observed were notable for occurring in testes that were profoundly atrophic. Both KS patients with TGCT had testicular volumes < 4 mL (well below the normal ∼15–20 mL per testis in adult males) and had azoospermia. Semen analysis demonstrated that 95.6% (108/113) of available samples confirmed azoospermia. This is consistent with previous studies providing data on azoospermia in adult KS cohorts, in which approximately 91%–96% of patients had azoospermia [19, 20]. Our study extends existing literature by situating azoospermia within a broader clinical profile.

In addition to testicular atrophy, other testicular abnormalities such as microlithiasis and hypoechoic lesions are also well documented in KS patients. In our cohort, 19 patients were found to have hypoechoic indeterminate lesions, one of which included the case of the midline tumour. Existing data indicate that the presence of both microlithiasis and hypoechoic lesions may predict worse testicular endocrine function and could play a role in the pathogenesis of TGCT and cryptorchidism [21].

Our study has some limitations: the KS cohort is moderate in size and from a single centre; although our incidence calculations were precise, a few cases here or there could change the point estimates. We also did not have a matched control group from the same time frame; instead, we relied on external registry data for comparisons, with cohort observation windows between 1943–1991 and 1979–2003 for the KS and non‐KS registries, respectively. These factors may affect comparability of incidence estimates and should be considered when interpreting relative‐risk results. However, we carefully chose comparative data from large population studies [4, 10] and included statistical analysis to account for person‐years. We were unable to calculate age‐specific incidence ratios for EGCTs due to the lack of published UK population‐specific rates. Crude SIRs were therefore calculated using Arora et al. (2012). Additionally, our cohort consisted of a Caucasian predominant sample given our monocentric study design which may not be reflective of the population as a whole. Given our imaging method was conventional B‐mode imaging with colour Doppler, we were unable to utilise the benefits of multiparametric ultrasound. Further studies involving new patients may shift towards multiparametric ultrasound to provide greater detail of testicular lesions [22]. We acknowledge that the incorporation if the paediatric cases may be a source of bias given the limited follow‐up time, however, we felt it was necessary to include as the comparative registries had also not omitted their paediatric cases from calculations. Another limitation is that our cohort's ascertainment of cryptorchidism may be incomplete (some older patients might not have documentation of an orchidopexy done decades ago, for instance). Nonetheless, our main conclusions are supported by multiple external studies (including registry and case series) beyond our own data. With respect to retrieval of data, semen analysis was not available for all 233 patients which may affect the overall proportion of patients with azoospermia in our cohort.

In summary, this study affirms that KS markedly increases the risk of extragonadal (mediastinal) GCTs but does not significantly elevate testicular cancer risk. Cryptorchidism in KS does not confer the same TGCT risk as in 46, XY males, likely due to early germ cell depletion and a unique hormonal and genetic environment. Understanding these mechanisms enhances clinical management and informs more nuanced surveillance strategies for individuals with KS.

Guy's and St Thomas’ NHS Foundation Trust audit registration number 18206.
